# Computational fluid dynamics simulation of airflow in the trachea and main bronchi for the subjects with left pulmonary artery sling

**DOI:** 10.1186/1475-925X-13-85

**Published:** 2014-06-24

**Authors:** Shouliang Qi, Zhenghua Li, Yong Yue, Han JW van Triest, Yan Kang

**Affiliations:** 1 Sino-Dutch Biomedical and Information Engineering School, Northeastern University, Shenyang, China; 2 Key Laboratory of Medical Imaging Computing (Ministry of Education), Northeastern University, Shenyang, China; 3 Department of Radiology, Shengjing Hospital of China Medical University, Shenyang, China

**Keywords:** Left pulmonary artery sling, Computational fluid dynamics, Airflow, Stenosis, MDCT

## Abstract

**Background:**

Left pulmonary artery sling (LPAS) is a rare but severe congenital anomaly, in which the stenoses are formed in the trachea and/or main bronchi. Multi-detector computed tomography (MDCT) provides useful anatomical images, but does not offer functional information. The objective of the present study is to quantitatively analyze the airflow in the trachea and main bronchi of LPAS subjects through computational fluid dynamics (CFD) simulation.

**Methods:**

Five subjects (four LPAS patients, one normal control) aging 6-19 months are analyzed. The geometric model of the trachea and the two main bronchi is extracted from the MDCT images. The inlet velocity is determined based on the body weight and the inlet area. Both the geometric model and personalized inflow conditions are imported into CFD software, ANSYS. The pressure drop, mass flow ratio through two bronchi, wall pressure, flow velocity and wall shear stress (WSS) are obtained, and compared to the normal control.

**Results:**

Due to the tracheal and/or bronchial stenosis, the pressure drop for the LPAS patients ranges 78.9 - 914.5 Pa, much higher than for the normal control (0.7 Pa). The mass flow ratio through the two bronchi does not correlate with the sectional area ratio if the anomalous left pulmonary artery compresses the trachea or bronchi. It is suggested that the C-shaped trachea plays an important role on facilitating the air flow into the left bronchus with the inertia force. For LPAS subjects, the distributions of velocities, wall pressure and WSS are less regular than for the normal control. At the stenotic site, high velocity, low wall pressure and high WSS are observed.

**Conclusions:**

Using geometric models extracted from CT images and the patient-specified inlet boundary conditions, CFD simulation can provide vital quantitative flow information for LPAS. Due to the stenosis, high pressure drops, inconsistent distributions of velocities, wall pressure and WSS are observed. The C-shaped trachea may facilitate a larger flow of air into the left bronchus under the inertial force, and decrease the ventilation of the right lung. Quantitative and personalized information may help understand the mechanism of LPAS and the correlations between stenosis and dyspnea, and facilitate the structural and functional assessment of LPAS.

## Introduction

**Figure 1:**
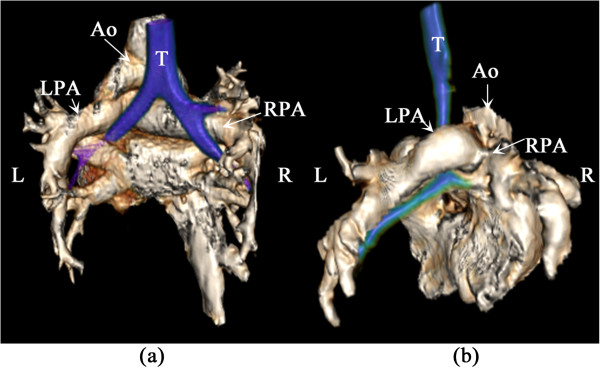
**Example of the normal and left pulmonary artery sling vasculature and airways in the posterior view (LPA – left pulmonary artery; RPA – right pulmonary artery; Ao – aorta; T – trachea; L – left; R – right). (a)** normal; **(b)** left pulmonary artery sling.

Left pulmonary artery sling (LPAS) is a rare congenital anomaly, where the left pulmonary artery (LPA) starts from the proximal portion of right pulmonary artery; initially runs to the right, passes behind the right main bronchus, turns left and then crosses between the trachea and esophagus, and finally reaches the hilum of the left lung [1,2]. A schematic representation of LPAS is shown in Figure 1. The anomalous LPA forms a “sling” around the distal trachea. Subjects with LPAS commonly present with respiratory problems resulting from external trachea and main bronchus compression. Surgeries including left pulmonary re-implantation with simultaneous repair of the tracheal stenosis are often carried out after diagnosis [3-5]. However, ideal surgery strategy is still controversial for there is no absolute correlation between luminal area of the stenosis and the prognosis of the disorder [3-5].

Multi-detector computed tomography (MDCT) has played an important role in the structural evaluation of congenital anomalies of the tracheobronchial tree and the lung [6]. The data however only gives information on the structural parameters of the lung, but no functional and quantitative information on the airflow can be measured.

Based on patient-specified geometric models extracted from the CT data, computational fluid dynamics (CFD) enables to reveal the nature of airflow in the trachea and bronchus tree under both physiological and pathology conditions [7,8]. Brouns et al. [9] found that the relationship between flow and pressure drop fits a power law, where the exponent is found to be 1.77, 1.92 and 2.00 for the absence of tracheal stenosis, 60% and 85% constriction, respectively. They suggested using the derived value for the exponent as a marker for stenoses at a pre-critical stage. Luo and Liu [10] simulated the respiratory flow in the airways up to 5 generations deep using a low Reynolds number (LRN) *k* − *ω* turbulent model. Deun [11] also employed this model to realistic CT derived lung geometry, and found an average pressure drop of 60.2 Pa for an inlet flow rate of 30 l/min. Freitas and Schroder [12] studied the steady flow in the upper human airways with the trachea and the sixth generation of the bronchial tree via a lattice-Boltzman method. In their work, the simulation is validated by particle-image velocimetry experiments.

Recently CFD simulations have been gradually accepted and adopted by the clinical medical society. Cebral and Summer [13] performed CFD simulations for four patients with Wegener’s granulomatosis using the tracheal and central bronchial models obtained by virtual bronchoscopy reconstruction. Low pressure and high shear stress were observed at the stenoses. Mihaescu et al. [14] investigated the flow in the pediatric airways with subglottic stenoses. Ho et al. [15] used CFD to investigate the airflow in the central airways before and after tracheobronchial stent implantation. They found that the CFD simulation results correlated well with those of pulmonary function tests (PFTs), indicating that CFD has the potential of being a clinical prognosis tool for the tracheobronchial stenosis. Mimouni-Benabu et al. [16] evaluated congenital tracheal stenosis and suggested CFD as a new objective tool for surgical decision-making. Shih et al. [17] reported the significant decrease of pressure drop, from 77.23 to 7.05 Pa after stent implantation for a 30.0 L/min airflow rate. Chen et al. [18] simulated the airflow in the trachea before and after the vascular ring surgery for 12 patients, and found that the pressure drop after surgery decreased significantly. More importantly, the accuracy and feasibility of CFD have been validated by hyperpolarized ^3^He magnetic resonance phase-contrast velocimetry [19], and SPECT/CT [20].

Meanwhile, the fluid-structure interaction (FSI) method has been applied to investigate the trachea and bifurcations including both realistic synthetic models and models extracted from CT images. Koombua and Pidaparti [21] analyzed the flow in the idealistic bronchial tree with 3, 4 and 5 generations; they observed that the tissue flexibility influenced the velocity, wall pressure, and wall shear stress about 2%, 7%, and 6%, respectively. Wall and Rabczuk [22] compared CFD and FSI simulations for the flow in the lower airways extracted from CT images, and found that the flow patterns are quite different although the changes of the cross section are small (about 2%). Xia et al. [23] investigated the flow in a section of the third and fourth generation bronchioles and found the peak wall shear stress for compliant airway wall was five times higher than that for rigid wall shear. Malve et al. [24] studied the tracheal deformability after the endotracheal stent implantation using a FSI method, and proposed CFD as a way to evaluate the predisposition of the stent to migrate.

It can be seen that CFD based on patient-specified geometric models extracted from CT imagery can combine structural and functional assessment of the airways. However, there are not many reports on CFD for LPAS patients, especially for the infantile patients. In this study we extract the trachea and main bronchi from the CT images, export to commercial available CFD software (*ANSYS Fluent 14.5,* ANSYS Inc., Pennsylvania, USA), and calculated the velocity, wall pressure and shear stress for four LPAS patients and one normal control. The objective is to quantitatively analyze the airflow and explore the new parameters of the inspiration function for the planning of a treatment strategy for patients suffering from LPAS.

## Material and methods

### Patients and CT data

CT images of four LPAS patients (three female, one male) and one healthy control (male) scanned from 2012 to 2013 are selected out of a database of Shengjing Hospital, China Medical University (Shenyang, China) for a retrospective study. The age of the five subjects ranges from 6 to 19 months. Scanning parameters include a tube potential in the range 80-120 kVp, slice thickness 0.5-2.0 mm, and pixel spacing 0.254-0.420 mm. All images are reconstructed in a 512 × 512 matrix. Details on the subjects and CT acquisition parameters are listed in Table 1. In the remainder, the healthy normal control will be indicated as A(H), and the other four LPAS patients are presented as B, C, D and E.

**Table 1:** Subject and CT acquisition parameters

**Subject**	**Gender**	**Age (month)**	**Weight (kg)**	**CT acquisition parameters**		
				**Tube potential (kVp)**	**Slice thickness (mm)**	**Pixel spacing (mm)**
A(H)	M	11	10.0	100	1.0	0.420 × 0.420
B	F	6	7.7	100	1.0	0.386 × 0.386
C	F	10	10.0	120	2.0	0.412 × 0.412
D	M	9	9.0	100	0.5	0.344 × 0.344
E	F	19	10.0	80	0.5	0.254 × 0.254

### CFD procedures and boundary conditions

The CT images are imported into an image processing platform, *Mimics 11.0* (Materialise, Leuven, Belgium). The trachea and main bronchi are extracted by a simple threshold segmentation algorithm combined with split and merge operations. After smoothing, the geometric model is transferred to a finite element module for surface meshing, and then is exported in Standard Tessellation Language (STL) format.

The STL mesh is transferred to a solid model through *SolidWorks 11.0* (SolidWorks Corp., Massachusetts, USA) and read into *ANSYS Fluent 14.5* (ANSYS Inc., Pennsylvania, USA). Tetrahedron elements and a patch independent algorithm are used during the meshing process. The base size of the mesh is 0.2 mm, but the final size is determined through mesh-independent evaluation with a tolerance of < 0.3%. Mesh quality is evaluated by the skewness as done by de Rochefort et al. [20]. For all cases, the maximum skewness ranges from 0.72 to 0.78, fulfilling the recommendation given by *ANSYS*. Depending on the size of the model, 400,000 to 1,400,000 elements are included.

For neonates, the tidal volume is estimated to range 5-7 mL/kg, and the respiratory rate is set to be 30-50 breaths/min [25]. The inspiration/expiration ratio is usually 1:2 or 1:3. Here we set the tidal volume of 5 mL/kg, the respiratory rate of 50 breaths/min and the inspiration/expiration ratio of 1:2 considering the patients are in respiratory distress with different severities. Hence, the inhalation rate and inlet velocity can be determined with known weight and inlet area, as shown in Table 2. It can be seen that the average inlet velocity ranges 4.14-4.77 m/s, and the Reynolds Number (*Re*) is in the range of 1542-1886 for subjects A(H), B, C and D. Because *Re* is less than 2000, a laminar model is selected for the simulations. Subject E has a severe segmental tracheal stenosis which makes the sectional area of the trachea as small as 6.00 mm^2^ (i.e., with a diameter of 2.8 mm). Correspondingly the inlet velocity reaches value as high as 20.83 m/s, and *Re* can reach up to 3942. Even though it may not be very realistic in this setting, the calculation is still done using the *k* − *ϵ* turbulence model. The inlet velocity for subject E is decreased gradually to study the airflow alterations. The two main bronchi outlets are set at a constant pressure of 1.0 atm.

**Table 2:** **Calculated inlet velocities and *Re *** **based on the patients’ weight, tidal volume and inhalation time from the reference**[25]

**Subject**	**Tidal volume (mL)**	**Inhalation time (s)**	**Inhalation rate (mL/s)**	**Inlet area (mm**^**2**^**)**	**Average velocity (m/s)**	***Re***
A(H)	50	0.4	125.0	29.18	4.28	1788
B	38.5	0.4	96.25	23.26	4.14	1542
C	50	0.4	125.0	26.21	4.77	1886
D	45	0.4	112.5	26.58	4.23	1686
E	50	0.4	125.0	6.00	20.83	3942

The wall of the airway is assumed to have a no-slip boundary. The air is assumed as a Newtonian fluid with a constant density of 1.225 kg/m^3^ and viscosity of 1.8 × 10^−5^ kg/m-s, respectively. These assumptions are reasonable as the pressure in the airway is rather low. A solution-vector norm of <10^−5^ is taken as the convergence criterion, and the pressure-velocity relation is considered to be coupled.

## Results

### Structural features, pressure drop and mass flow rate

**Figure 2:**
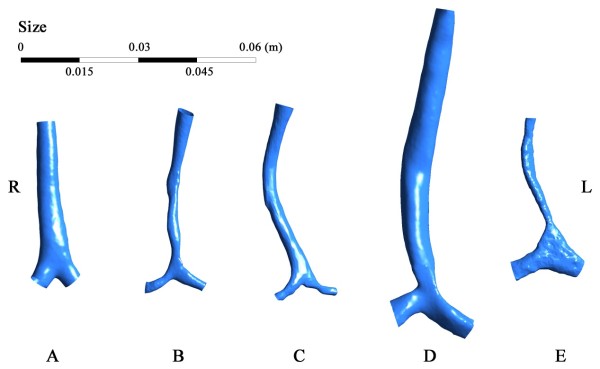
Tracheal models of the subjects studied (R – right, L – left; A – E: five subjects).

The extracted models of the trachea and main bronchi are shown in Figure 2. Compared to the normal control, the tracheas of the subjects with LPAS follow a C-shaped curve due to the compression of the trachea by the LPA from the right-posterior side. For subjects B, C and E, a segmental stenosis is found, but a local stenosis is found near the tracheal distal end for subject D. Subject E is special due to the two big main bronchi and a narrow trachea.

Once the CFD simulations are done for all subjects, the average inlet pressure, outlet sectional area, minimum sectional area and mass flow rates are calculated and summarized in Table. 3. The pressure drop defined as

(1) $$\mathrm{\Delta P}=P_{\mathrm{inlet}}-\left(P_{\mathrm{outlet}1}+P_{\mathrm{outlet}2}\right)/2$$

is calculated to evaluate the flow resistance, where *P*_*outlet*1_ and *P*_*outlet*2_ are the pressure at right and left bronchus, respectively. Both *P*_*outlet*1_ and *P*_*outlet*2_ are equal to zero as they are set at a constant pressure of 1.0 atm. For the normal control, the pressure drop is only 0.68 Pa, but for the LPAS patients, it reaches up to 78.90-914.50 Pa to maintain normal physiology, explaining why dyspnea and stridor usually accompanying LPAS.

**Table 3:** Calculated inlet pressure, outlet pressure and mass flow rate

**Subject**	**Inlet pressure (Pa)**	**Outlet cross-sectional area (mm**^**2**^**)**		**Outlet minimum cross-sectional area (mm**^**2**^**)**		**Outlet mass flow rate × 10**^**6 **^(kg/s)*		**Outlet flow velocity (m/s)**	
		**Left**	**Right**	**Left**	**Right**	**Left**	**Right**	**Left**	**Right**
A(H)	0.68	24.26	27.70	24.26	27.70	−71.54	−81.59	2.52	2.65
B	475.98	4.30	7.19	4.30	4.26	−44.31	−73.62	8.81	8.61
C	282.81	5.17	5.50	2.72	5.43	−51.04	−102.02	8.43	15.32
D	78.90	15.73	26.49	15.73	18.09	−118.51	−19.32	6.39	1.46
E	914.50	13.63	15.74	13.63	15.74	−216.03	66.32	14.14	3.44

*For mass flow rate, minus means flow-out and positive is flow-in.

The ratios of the cross-sectional areas of the left and right main bronchi are represented by

(2) $$R_{\mathrm{AL}}=SA_{L}/\left(SA_{L}+SA_{R}\right)$$

(3) $$R_{\mathrm{AR}}=SA_{R}/\left(SA_{L}+SA_{R}\right)$$

where *SA*_*L*_ and *SA*_*R*_ are the cross-sectional area of left and right main bronchi, respectively.

Correspondingly, the ratios of the minimum cross-sectional area of the left and right main bronchi are given as

(4) $$R_{\mathrm{MAL}}=\mathrm{MS}A_{L}/\left(\mathrm{MS}A_{L}+\mathrm{MS}A_{R}\right)$$

(5) $$R_{\mathrm{MAR}}=\mathrm{MS}A_{R}/\left(\mathrm{MS}A_{L}+\mathrm{MS}A_{R}\right)$$

where *MSA*_*L*_ and *MSA*_*R*_ are the minimum sectional area of left and right main bronchi, respectively. Moreover, the ratios of the flow rate are defined as

(6) $$R_{\mathrm{FL}}=FR_{L}/\left(FR_{L}+FR_{R}\right)$$

(7) $$R_{\mathrm{FR}}=FR_{R}/\left(FR_{L}+FR_{R}\right)$$

where *FR*_*L*_ and *FR*_*R*_ are the outlet flow rate for left and right main bronchi.

**Figure 3:**
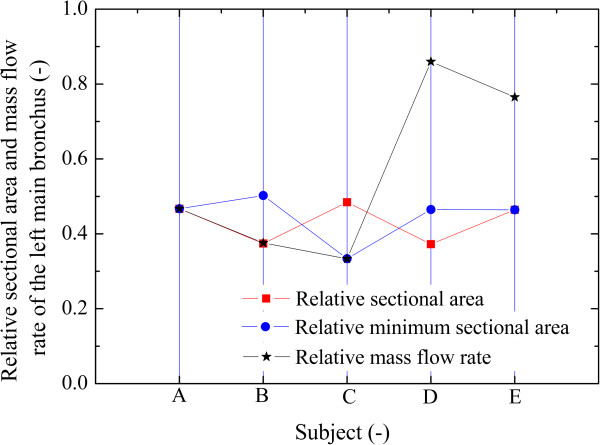
The cross sectional area and mass flow rate of the left main bronchus.

Estimated values for *R*_*AL*_, *R*_*MAL*_ and *R*_*FL*_ are given in Figure 3 for comparison. It is found that *R*_*AL*_ is less than 50% (37.24 - 48.45%) for all subjects. For the normal control A(H), *R*_*AL*_ (46.69%) and *R*_*FL*_ (46.72%) are almost equal. This results from the fact that the the airflow in the two bronchi have nearly equal average velocity. For subject B, *R*_*FL*_ and *R*_*MAL*_ are also equal. For subject C, a stenosis at the left main bronchus results in a low *R*_*FL*_ of 33.34%. For subjects D and E, however, there is no correlation between *R*_*FL*_ and *R*_*AL*_ (or *R*_*MAL*_) as the trachea or bronchus is compressed. For subject D and E, *R*_*FL*_ is 85.98% and 76.51%, larger than 50% though *R*_*AL*_ <50%, which means cross-sectional area is not the only dominating parameter. The C-shaped curve of the trachea may play an important role, and facilitate the airflow to the left bronchus by means of inertia. This point will be discussed for subject E later. The found lack of correlation between flow and sectional area indicates that CFD may provide a new functional evaluation for LPAS, which normal structural imagery cannot offer.

### Wall pressure, velocity and wall shear stress

#### Normal control

**Figure 4:**
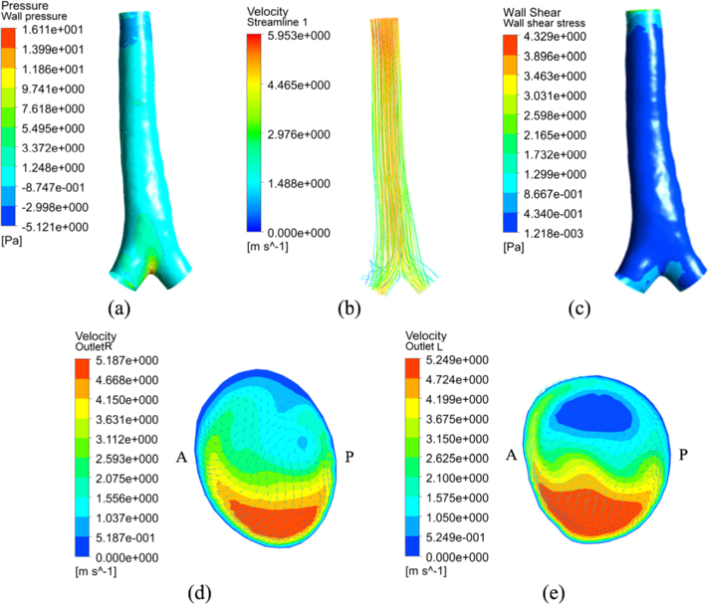
**Calculated results for the normal control subject A(H). (a)** wall pressure; **(b)** velocity streamlines; **(c)** wall shear stress; **(d)** velocity profile at right main bronchus; **(e)** velocity profile at left main bronchus.

For the normal control (subject A(H)), the calculated results, including wall pressure, velocity streamline, wall shear stress (WSS) and velocity profiles at both main bronchi are shown in Figure 4. The wall pressure is distributed rather evenly, and attains a maximum point of about 16 Pa at the bifurcation due to the direct impact of the airflow. Correspondingly no significant variation appears for the velocity and WSS. For the bronchi, the high speed regions are at the bottom as shown in (d) and (e) of Figure 4. As given in (c) of Figure 4, the WSS is also high at these places. The flow pattern at the right bronchus agrees well with past numerical [10] and experimental results [20], though the absolute velocities are dissimilar for the different geometric models.

#### Subject B, C and D

**Figure 5:**
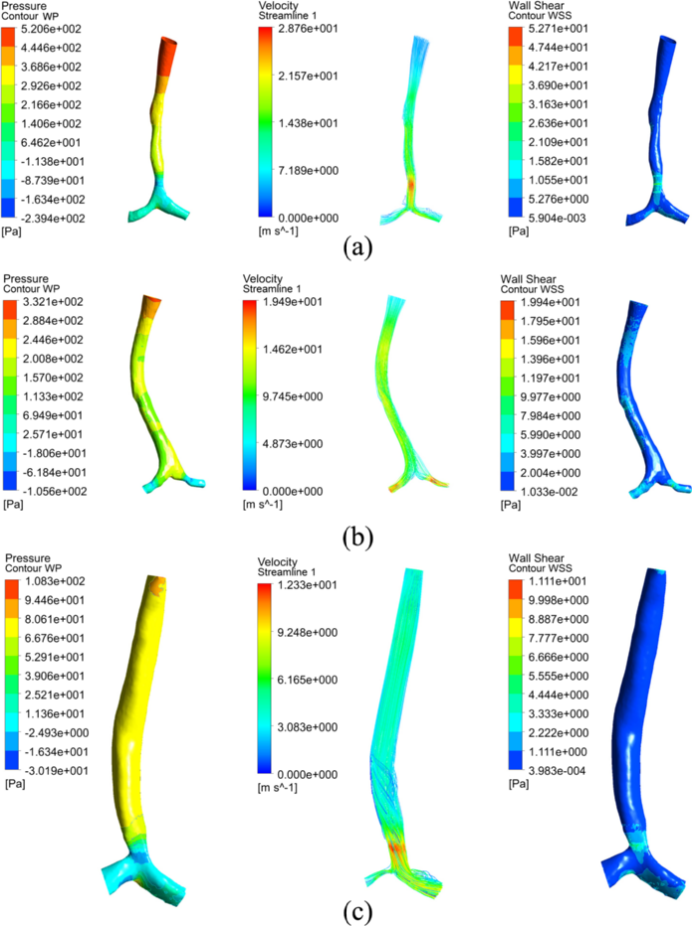
**Calculated wall pressure, velocity and wall shear stress for different patients. (a)** subject B; **(b)** subject C; **(c)** subject D.

The wall pressure, velocity and WSS are patient-specified as shown in Figure 5, but some generalizations can be made. Firstly, the distributions of the three parameters are more variable and inconsistent than for the normal control. Secondly, the maximum wall pressure appears at the inlet, which differs with the normal control. This is because the stenosis increases the flow resistance and inlet driven pressure. The values (521, 332 and l08 Pa for subject B, C and D) are far higher than that of the normal control (16 Pa). Thirdly, the low wall pressure, high velocity and high WSS are all found at the stenosis regardless whether the stenosis is at the trachea or the bronchi. The maximum velocities (28.8, 19.5 and 12.3 m/s for subject B, C and D) and wall shear stresses (52.7, 19.9 and 11.1 Pa for subject B, C and D) are located at the stenosis, and their values are higher than in the normal control (6.0 m/s and 4.3 Pa respectively), increasing with the stenosis severity.

For subjects B-E, negative pressures of -103.5, -15.1, -8.16 and -518.9 Pa are found at the stenosis.

#### Local features

**Figure 6:**
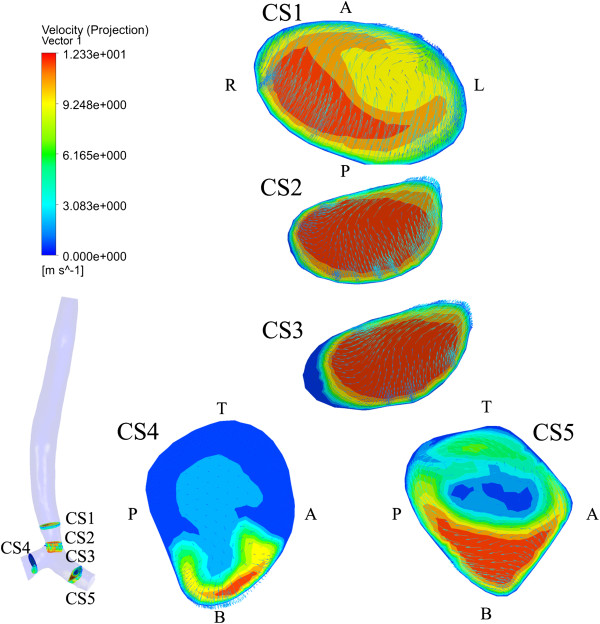
The local velocity profile for subject D (A – anterior; P – posterior; T – top; B – Bottom; R – right; L – left).

Besides the global flow patterns, CFD simulations can also provide a detailed local velocity profiles. The results of subject D are shown in Figure 6 as an example. Totally five cross sections are selected. Cross-sections CS1 and CS3 are before and after the stenosis, CS2 is located at the tracheal stenosis, and CS4 and CS5 are at the right and left main bronchi, respectively. It is found that the stenoses and post-stenotic dilatation lead to acceleration and deceleration respectively. The cross-sectional areas at CS1, C2 and CS3 are 22.45, 11.22 and 11.79 mm^2^, and the average velocities are 5.41, 10.27 and 9.80 m/s, respectively. For CS4 and CS5 with similar cross sectional areas, the average velocity is quite different, 1.53 and 5.06 m/s, respectively. The C-shape of the trachea can be given as a reasonable explanation, as mentioned before. At both CS4 and CS5, high velocity regions appear at the bottom which is similar to the normal control, but the profile is more irregular.

#### Subject E

Due to the severity of the stenosis, subject E is investigated separately. To maintain normal physiological conditions in the lungs, the inlet velocity should reach up to 20.83 m/s, which is impossible to achieve. The subject therefore died from the dysnapic apnea. Due to the severe tracheal stenosis, the bronchi and bifurcation were extremely inflated, as shown in Figure 2, to decrease the flow resistance as to meet with the lowest physiological needs.

**Figure 7:**
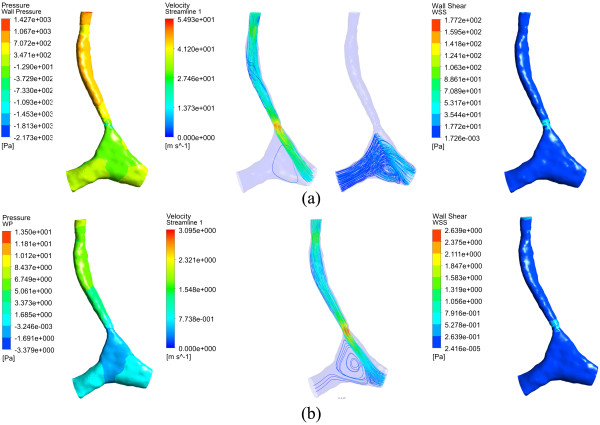
**Wall pressure, velocity streamlines and wall shear stress for subject E at different inlet velocities. (a)** 20.0 m/s; **(b)** 1.0 m/s.

From this special case, the role of the C-shaped trachea can be further understood. As show in (a) of Figure 7, no air flows out from the right main bronchus even though the necessary anatomy is available at the inlet velocity of 20.83 m/s. On the contrary, some air with a mass flow rate of 6.63 × 10^−5^ kg/s flows in from the right main bronchus. A pressure of -7.6 Pa also shows that the air is directed inward from this bronchus. Meanwhile, the wall pressure and WSS reach unrealistic values of up to 1427 and 177 Pa.At the inlet velocity of 1.0 m/s, it is found that air flows out from the right main bronchus by a vortex-shaped streamline, as shown in (b) of Figure 7, explaining why the air has difficulty to flow out of the right main bronchus. The maximum wall pressure and WSS become 13.5 and 2.6 Pa in these situations.

**Figure 8:**
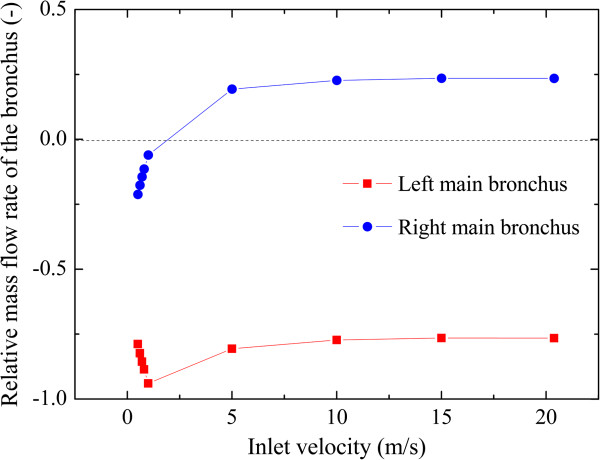
Relative mass flow rate in the bronchus with different inlet velocities for subject E.

It has been suggested that the C-shaped trachea makes the flow deviate to the right side due to the inertia. To decrease the role of inertia, the inlet velocity is dropped gradually from 20.83 to 0.5 m/s, and *R*_*FL*_ and *R*_*FR*_ are calculated for each of these experiments. As shown in Figure 8, *R*_*FR*_ is positive while the inlet velocity is larger than 1.0 m/s. At an inlet velocity of 1.0 m/s, *R*_*FR*_ turns negative, indicating the air flows out from the right main bronchus. Further decrease of inlet velocity from 1.0 to 0.5 m/s will increase *R*_*FR*_ from 6.0% to 21.2%.

## Discussions

For the CFD simulation of airflow in the respiratory tract, several important assumptions are made, including no-slip boundary conditions, modeling air as an incompressible Newtonian fluid with constant density and viscosity, and steady static pressure. These assumptions have been accepted and widely used [7-20,26]. The specified velocity inlet and pressure outlet boundary conditions are considered as a simple and feasible method [9,10,14-16,20,26]. The inlet velocity (or flow rate) is usually determined based on past experiences. For adults, the flow rate reported in literature varies widely, and takes values including 6 L/min [15], 30 L/min [19], 16.1 L/min [20], and 28.3 L/min [26]. In infants, the flow rate is smaller, and is set to values such as 3.5 and 10 L/min [14], 3.0 and 7.3 L/min [16]. In the present study, the inlet flow rate is individualized according to the weight, and ranges from 5.8 to 7.5 L/min. It is based on the tidal volumes, which is 5-7 mL/kg for a neonate and 7 mL/kg for an adult, the respiratory rate is 30-50 and 12-16 breaths/min [25], and the inspiration/expiration ratio 1:2 to 1:3.

For subject E, the *k* − *ϵ* turbulence model is applied for *Re* > 2000. Though this model was shown to tend to over-predict the pressure values, it has been used in several other studies [27]. For the other subjects, a laminar model is deemed realistic, as the Reynolds number ranges from 1542-1886, due to the lower inlet velocity and tracheal diameter in infants.

Pulmonary function tests (PFTs) are not feasible for infants aging 6-19 months, even though they are useful for the evaluation of the pulmonary function. CFD results have shown to be consistent with the PFTs for the study of the alternation of the airflow before and after trachea-bronchial stent placements [15]. It is suggested that CFD may offer an important approach to assess the pulmonary function for the patients unsuitable for PFTs.

There are many CFD studies focusing on clinical diseases, such as Wegener’s grnulomatosis [13], pediatric airways with subglottic stenoses [14], tracheal stent implantation [15,17], congenital tracheal stenoses [16], and stenoses caused by the vascular rings [18]. To the best our knowledge the airflow in the trachea and main bronchi of LPAS patients aged 6-19 month has not yet been investigated by means of CFD before. The geometry size and CFD boundary conditions are different from past studies. To compare the current study with those for the elder LPAS patients may be useful to know the development of this disease.

Normal human airways show high ventilation efficiencies. The pressure drop is only 0.68 Pa for the healthy infant, which is close to the 0.70_±_0.41 Pa for the healthy adult [15]. Even for the airway 17 generations deep in a healthy adult, the pressure drop is only 50.0 Pa while the flow rate is 28.3 L/min [26].

For the subjects with LPAS in this study however, the pressure drop ranges 78.9-914.5 Pa, mainly resulting from the tracheal and/or bronchial stenoses. The amount of pressure drop varies with the severity of the stenoses. Ho et al. [15] calculated a pressure drop of 10.61_±_9.22 Pa for the adult patients with tracheal stenosis while the flow rate is determined to be 6.0 L/min. For the congenital tracheal stenosis, the pressure drop is also found to reach up to 1825 Pa with a flow rate of 7.3 L/min. In fact, the maximum pressure difference between the alveoli and the outside atmosphere, which results from the movement of diaphragm and intercostal muscles, is about 94 Pa to sustain an inspiration of 30 L/min [28]. Therefore, extremely high pressure drops indicate that the flow rate cannot meet the minimum requirement for normal physiology, and the stridor, dyspnea, and even death from suffocation (e.g., subject E) will occur.

The ratios of inflow between the left and right lobes are 46.72% and 53.28% for the normal control. Other results reported in literature are 43.43% and 56.57% for the average of 7 healthy adult subjects [15], 48.2% and 51.8% for the average of 6 patients with mild asthma [19], and 44% and 56% in a 60-years-old Chinese male patient [26]. The ratios of cross sectional area for the left and right bronchi are 46.69% and 53.31%, practically the same as the inflow ratios. For subject B, similar results are found.

Neither *R*_*MAL*_ nor *R*_*AL*_ can determine *R*_*FL*_ independently; the shape of trachea must also be considered. As shown in Figure 3, *R*_*FL*_ accords with *R*_*AL*_ for subject B, but accords well with *R*_*MAL*_ for subject C. For subject D and E however, the ratios of area and inflow are significantly different. For subjects D and E, the area ratio of left bronchus is 37.26% and 46.41% respectively, but the flow ratio reaches up to 86.98% and 76.51% respectively. This phenomenon is explained by the C-shaped trachea caused by the anomalous LPA. Due to the inertia, a large fraction of the air flows into the left bronchus. This point is demonstrated by two aspects. Firstly, for subject E with an inlet velocity of 20.3 m/s, the right bronchus even experiences negative pressure, and thus air flows out of it. Secondly, the inertia is further visible by decreasing the inlet flow, i.e., the inertia force for subject E. As the inertia force drops, the inflow from the right main bronchus turns into an outflow at the inlet velocity of 1.0 m/s, and the ratio of flow to the right reaches to 21.2% for a 0.5 m/s inlet velocity.

The above observations indicate that the right bronchus (or lung) can be reached anatomically, but it does not play any role during inspiration. The C-shaped trachea may lead to the functional “hypogenesis” of the right lung. To the best of the authors’ knowledge, these findings have not been reported in previous studies. Moreover, it demonstrates that CFD provides vital functional information which CT structure images cannot offer.

For subjects B-E, negative pressures of -103.5, -15.1, -8.16 and -518.9 Pa are found at the stenoses, respectively. A study on subglottic stenoses also observed a negative pressure of -845 Pa [14]. The negative pressure is dangerous as the possible collapse of the malacic segment may occur at the stenotic site.

The WSS indicates the forces exerted tangential to the inner luminal wall of the airway. At laminar flow for the normal control, the WSS is small due to the developed steady boundary layer. In the afflicted subjects however, airflow is accelerated leading to turbulence which disrupts the boundary layer and results in high WSS. As large WSS variations in blood vessels may increase the risk of arteriosclerosis, the high WSS at the stenotic site is correlated with the trauma of the airway mucosa and an inflammatory response [14].

Limitations for the present study should be pointed out as well. Firstly, the number of subjects in this study is very small, only including four patients and one normal control subject. Secondly, the CFD results are not validated by the experimental measurement as done by [20,27] though observed flow patterns are in close agreement with numerical and experimental results [10,20]. Thirdly, only the trachea and two main bronchi are modeled as the stenoses usually present at these locations. However, to include the bronchi of higher generations might give more information. Fourthly, the steady state analysis for inspiration is adopted. Transient study including both inspiration and expiration, however, will reveal more details. Finally, both trachea and bronchi demonstrate a large Young’s modulus (1.31 MPa [21], 9.00 MPa [22]) owing to the availability of cartilage, and as such the deformability might be small. The airway is assumed to have a rigid wall, and the interactions between airways and airflow are not considered. In fact, the flow velocity, airway pressure and WSS might be affected though the cross sections of the trachea and bronchi do not change significantly [21-24]. In further work, the authors intend to study the effects of the above limitations carefully.

## Conclusions

Using geometric models extracted from CT images and patient-specified inlet boundary conditions, CFD simulation can provide vital airflow information including flow features and the quantitative parameters for LPAS. Due to the tracheal and/or bronchial stenosis, a much higher pressure drop is found for LPAS patients. The C-shaped trachea may facilitate a higher air flow into the left bronchus under the inertial force, which means the ventilation to the right lung may be decreased, even though its anatomic structure remains normal. Besides the qualitative finding that high air velocity, low wall pressure drop and high WSS occur at stenotic sites, more quantitative and personalized information can be obtained. These results may help understand the mechanism of LPAS and the correlations between stenosis and dyspnea, and facilitate the structural and functional assessment of LPAS.

## Abbreviations

CFD: Computational fluid dynamics; FSI: Fluid-structure interaction; LPA: Left pulmonary artery; LPAS: Left pulmonary artery sling; MDCT: Multi-detector Computed Tomography; PFTs: Pulmonary function tests; STL: Standard tessellation language; WSS: Wall shear stress.

## Competing interests

The authors declare that they have no competing interests.

## Authors’ contributions

SQ: proposed the idea, performed experiments, analyzed the data, made discussions and composed the manuscript together with ZL, HJWT. YY: provided CT images and radiology instruction, and made the discussions. YK: directed the experiments and made discussions. All authors read and approved the final manuscript.
